# Green synthesis of nanoselenium by *Ferula assa-foetida* (L.) root extract: Docking study, antimicrobial activity, and its role as a biostimulant in *Vicia faba* (L.) seedlings

**DOI:** 10.1186/s12870-025-06954-4

**Published:** 2025-07-19

**Authors:** Eman Zakaria Ahmed, Amira Mohamed Abd El-Sattar, Eslam T. Mohamed, Muhammad Gamal Abdelmaksoud, Heba El-Sayed

**Affiliations:** 1 Botany and Microbiology Department, Faculty of Science, Helwan University, Helwan, 11795 Egypt; 2 Molecular Biotechnology program, Faculty of Science, Helwan University, Cairo, 11795 Egypt

**Keywords:** *Ferula assa-foetida* (L.), Green synthesis, SeNPs, Antimicrobial, Antioxidant activity, *Vicia faba* (L.) growth

## Abstract

**Background:**

Green synthesis of selenium nanoparticles (SeNPs) using *Ferula assa-foetida* L. root extract offers an eco-friendly approach for biomedical and agricultural applications by leveraging plant metabolites. This study characterized SeNPs, evaluated antimicrobial and antioxidant properties of the extract and SeNPs, assessed SeNPs’ biostimulant effects on *Vicia faba* L. seed germination, and explored bioactivity mechanisms via molecular docking.

**Results:**

SeNPs, averaging 90.4 nm, were characterized using UV-Vis spectroscopy, Fourier transform infrared (FT-IR) spectroscopy, transmission electron microscopy (TEM), zeta sizer, and zeta potential. SeNPs exhibited notable antimicrobial activity against *Escherichia coli*, *Staphylococcus aureus*, and *Candida albicans*. Inhibition zones were: *E. coli* (SeNPs: 18 mm; extract: 15 mm), *S. aureus* (SeNPs: 15 mm; extract: 12 mm), and *C. albicans* (SeNPs: 13 mm; extract: 17 mm). Molecular docking linked these to SeNPs’ binding to *E. coli* RNA polymerase (3LU0, -3.3224 kcal/mol), *S. aureus* RNA polymerase (4EDG, -3.0732 kcal/mol), and *C. albicans* CYP51 (5FSA, -3.5097 kcal/mol), indicating transcription and fungal membrane disruption. The extract exhibited strong antioxidant capacity via DPPH assay (IC_50_: 37.38 µg/mL), while SeNPs showed moderate activity (IC_50_: 338.4 µg/mL), correlated with superoxide dismutase binding (2SOD, -2.0912 kcal/mol), enhancing radical scavenging. Seed priming with low SeNP concentrations (1 and 5 µM) increased *Vicia faba* shoot length (56.66%, 30%) and root length (4 to 8.5 cm at 5 µM), with elevated sugars (57.06% at 1 µM) and proteins (33.1% at 1 µM), tied to auxin receptor TIR1 binding (2P1Q, -3.3108 kcal/mol), promoting growth signaling.

**Conclusion:**

*Ferula assa-foetida* extract and SeNPs demonstrated antimicrobial efficacy against multidrug-resistant pathogens, with the extract’s phytochemicals supporting green SeNPs synthesis. SeNPs outperformed the extract against *E. coli* and *S. aureus*, while the extract excelled against *C. albicans*. Low SeNP concentrations enhanced *Vicia faba* growth, promising for agriculture, but high doses inhibited growth, requiring caution. Molecular docking suggested mechanisms needing validation. Further phytochemical studies were recommended for medical and agricultural applications, pending clinical testing.

## Background

Considerable research has been undertaken on the natural compounds derived from plants, both in terrestrial and aquatic environments. Historically, plant-based therapies have been employed to address and prevent various diseases due to the diverse biological properties of plant natural products, such as antioxidant, anti-inflammatory, antibacterial, antifungal, antiparasitic, analgesic, antidiabetic, anti-atherogenic, and antiproliferative effects [1]. The escalating mortality rate caused by antibiotic-resistant bacteria has emerged as a worldwide health issue, often attributed to the improper use of antibiotics that may result in antibiotic resistance. One approach to tackle these issues is by using natural agents, such as active chemicals derived from plants [2, 3].

The genus *Ferula* (family Apiaceae), which is distributed throughout much of central Asia, North Africa, and the Mediterranean region, contains more than 170 species [4]. Assa-foetida is a natural gum composed of oleoresin derived from the stem and rhizome of the *Ferula assa-foetida* plant. Hing or Devil’s Dung are two colloquial terms used to refer to it. The natural geographical distribution of this species includes Iran, Afghanistan, and Pakistan [5]. It can withstand dryness and little rainfall since it is well suited to dry environments. Additionally, it grows well in soils that include a lot of sand [6]. The scent is sulfurous, strong, and potent. It is extensively used as a seasoning for curries, fresh vegetables, meat, pickles, and pulses, as well as a spice or condiment in many culinary dishes.

The plant possesses several pharmacological and therapeutic properties, such as antioxidant, antibacterial, antifungal, antiviral, anticancer, antidiabetic, antispasmodic, hypertensive, hepatoprotective, and neuroprotective effects [5]. Historically, the herb has been utilized for the treatment of several ailments such as influenza, impaired digestion, whooping cough, asthma, bronchitis, epilepsy, ulcers, stomachaches, and flatulence. The principal bioactive components present in *Ferula assa-foetida* plants include essential oils, gums, and resins. Additionally, *Ferula assa-foetida* contains a diverse range of chemical constituents, including minerals, carbohydrates, moisture, protein, and fats [7]. Iranshahy and Iranshahi [8] identified resins (40–64%), gums (25%), and essential oils (10–17%) as the primary components comprising the principal chemical additives. The *F. assa-foetida* plant also includes diterpenes and phenolic chemicals, including vanillin, 3,4 dimethoxycinnamyl-3-(3,4-diacetoxyphenyl) picealactone C, and 7-oxocallitristic acid (4-oxocallitristic acid) [9], coumarin, coumarin esters, sesquiterpenes, sesquiterpene lactones, monoterpene, monoterpene coumarins, prenylated coumarins, flavonoids, in addition to sulfur-containing compounds, phytoestrogen [10].

Nanotechnology is a highly influential and rapidly growing field within the domains of science, technology, health, and agriculture [11]. The utilization of natural resources is an environmentally sustainable method for manufacturing biomaterials at the nanoscale, ranging from 1 to 100 nm [12, 13]. Their higher surface-to-volume ratio and elevated surface energies render them particularly valuable for a diverse array of biological applications [14]. Nanoparticles (NPs) are widely manufactured and utilized particles because of their exceptional characteristics, such as their capacity to be optically polarized, compatibility with living organisms, and their antioxidant, antibacterial, and catalytic behavior [15, 16].

Nanoparticle synthesis utilizing biomaterials has garnered significant interest due to its unique characteristics, such as cost-effectiveness, straightforward synthesis procedures, strong water solubility, and environmental friendliness [17, 18]. Reliable, simple, safe, cost-effective, and ecologically advantageous methods are provided by natural organisms, microorganisms, microalgae, enzymes, plants, and plant extracts [19, 20].

Non-metal selenium (Se) is an essential micronutrient for optimal physiological and metabolic functions in humans and other animals [21]. The recommended daily intake of selenium for an average adult is between 40 and 300 grammes through dietary supplements. Selenium is employed as a cofactor in several enzymatic reactions and is present in animals as proteins that encode selenium [22]. Nanoparticles of elemental selenium have recently garnered significant interest because of their distinct physical and chemical characteristics that deviate from those of the comparable bulk materials [23]. Nano-Se, a bright red, soluble, extremely stable, and nano-sized material in the redox state of zero (Se^0^), has been produced for use in nutritional supplements and has also been developed for medical therapeutic applications [24]. Given its excellent bioavailability, antioxidant activity, antibacterial capabilities, and low toxicity, selenium nanoparticles (SeNPs) are among the many types of nanoparticles that are generating significant attention [25, 26]. According to Chen et al. [27], it also exhibits a more potent therapeutic effect on cancer.

The conventional approach for biosynthesizing SeNPs is to introduce microorganisms or plant extracts into the sodium selenite or selenate solution. A range of biomolecules present in plant extracts, such as polysaccharides, phenolic compounds, flavonoids, tannins, saponins, enzymes, proteins, and sugars, have been recognized as potential serum selenium reducing agents with potential therapeutic applications. Typically, these substances are used as bio-reductants in the production of nanoparticles [28]. Shi et al. [29] synthesized *Lycium barbarum* polysaccharide-capped selenium nanoparticles (SeNPs) at ambient temperature by utilizing green tea extract as a reducing agent.

The biosynthesized SeNPs are characterized by high activity, tiny particle size, rugged stability, high temperature resistance, and uniform size. Given their established antibacterial properties, SeNPs have the potential to serve as a viable substitute for antibiotics by disrupting bacterial structure and preventing multidrug-resistant bacterial infections [30]. Nile et al. [31] found that biogenic SeNPs exhibited a potent inhibitory impact on *Aspergillus fumigatus* and *Candida albicans*. Joshi et al. [32] presented evidence that myogenic SeNPs exhibited effective antifungal properties against *Colletotrichum capsici* and *Alternaria solani*. This finding emphasizes the potential of employing Se NPs for the environmentally friendly treatment of plant diseases. Nano selenium has been documented for its efficacy in promoting and stimulating agricultural growth. Based on the study conducted by Sariñana-Navarrete et al. [34], the application of nano-selenium at low concentrations to *Capsicum annuum* L. seeds resulted in enhanced root length, germination percentage, germination rate, and seed vigor in the seedlings. Conversely, high doses of selenium nanoparticles suppressed seed germination.

Unlike commonly studied plants for SeNPs synthesis, such as *Allium sativum* or *Lycium barbarum*, *Ferula assa-foetida*’s root extract remains underutilized, offering a novel approach due to its distinct chemical composition, which may enhance SeNP stability and efficacy [35]. The integration of *Ferula*-mediated SeNP synthesis with antimicrobial, biostimulant, and molecular docking analyses represented a unique framework to explore its multifaceted bioactivity, distinguishing this study from prior confirmatory studies.

The aim of this work was to assess the antimicrobial and antioxidant properties of *Ferula assa-foetida* L. plant extract and selenium nanoparticles biosynthesized using the same plant extract. Furthermore, use of SeNPs on *Vicia faba* (L.), a highly significant crop in Egypt, during the initial stages of seedling development.

## Materials and methods

### Plant material

Roots of *Ferula assa-foetida* purchased from the local market in Saint Catherine, Sinai, Egypt in 2019. The dried gum of the root was ground into a fine powder and preserved for subsequent use.

### Extraction of dry plant

A *Ferula assa-foetida* root aqueous extract was prepared according to the method described by Niazmand and Razavizadeh [36] with some modifications using deionized distilled water. Five grams of air-dry root gum were extracted in 100 ml water at 60 °C for an hour with continuous stirring. Clear solutions were obtained by centrifuging the extract for 15 min at 4000 rpm after filtration through Whatman filter paper No. 1.

### Preparation of nano-selenium

Each 50 ml plant extract at pH 6.5 was supplemented with one gram of sodium selenite and then incubated at 40 °C for two days. The formation of nano selenium particles was confirmed by the transformation of the solution color from colorless to a dark orange color. Before characterizing the nanoparticles, the mixture was subjected to five cycles of centrifugation, during which the pellets were rinsed with deionized water [37] to remove plant extract residue. Selenium nano particles were dried at 40 °C and stored in dry conditions for characterization and different biological applications.

### Characterization of nano-selenium

Selenium nanoparticles were characterized by several techniques to confirm their production and ascertain their size, structure, and stability. The produced SeNPs absorption peak was characterized using a T80 + UV/VIS Spectrometer PG Instruments Ltd at Bacteriology lab Faculty of Science– Helwan University in Egypt over the wavelength range of 200–800 nm.

The Fourier Transform Infrared (FTIR) spectra of SeNPs were obtained using the KBr Pellet technique at the Central Lab. Faculty of Science– Helwan University in Egypt. The spectra were recorded at a resolution of 2 cm^−1^ inside the wave numbers range of 400–4000 cm^−1^. In this study, the zeta potential was determined using a Zeta sizer Nano ZS particle size analyzer from Malvern Instruments, UK. The dynamic light scattering approach was employed at a temperature of 25 °C utilizing the PSS-NICOMP 380-ZLS particle sizing system. At Central Lab, the precise dimensions and structure of the nanoparticles were observed using the JEOL electron microscope JEM-100 CX (JEM-2100 h) model at the Faculty of Agriculture, Cairo University in Egypt.

### Total antioxidant activity

The scavenging effectiveness of the extract and the biosynthesized Se-NPs against DPPH radicals was evaluated at different dosages. The reaction was conducted at ambient temperature for 30 min in the absence of light, using 100 µl of recently condensed DPPH reagent (0.1% in methanol) added to 100 µl of the samples in a 96-well plate (*n* = 6). Quantification of the resulting reduction in DPPH color intensity at a wavelength of 540 nm. The data are presented as means ± standard deviation as calculated using the following formula. Subsequently, the IC_50_ value for the selenium nanoparticles was determined.

### *Invitro* antimicrobial activity

In order to assess the antimicrobial activity of both the natural plant extract and the biosynthesized SeNPs, pathogens including *Staphylococcus aureus*, *Candida albicans*, and *Escherichia coli* were graciously given by the Pharmaceutical Control Authority, Giza, Egypt. For the production of bacterial and yeast seeded plates, 100 µl suspensions of each culture under investigation were introduced onto nutritional agar medium. To form wells on the agar surface, a 6-mm cork borer was employed. In order to reach a final concentration of 20 µg/ml, the produced selenium nanoparticles was dissolved in sterile distilled water. One hundred microliters of the tested sample were individually dispensed into each well using a sterile syringe. Following the diffusion of the extract into the agar, the plates treated with the seed were incubated for 24 h at a temperature of 37 ± 2 °C. The plates exhibited the obvious formation of the inhibition zone encircling the wells. The diameter of the inhibition zone around the well, measured in millimeters, was calculated by taking into account the diameter of the well in three corresponding replicas. After measuring each plate in three specified fixed directions, the average values for the three independent replicas were tabulated [38]. Vancomycin and gentamicin were utilized as typical antibacterial agents, while amphotericin B served as the standard antifungal agent. The negative control consisted of a well containing 100 µl of distilled H_2_O.

### Se-nanoparticle effect on *V. faba* (L.) plant growth

Seeds of *V. faba* (L.) (Broad bean) were generously supplied by the Agriculture Research Centre in Cairo, Egypt. Selection of uniform seeds was followed by immersion in biosynthesized nano selenium solution at various concentrations (1, 5, 10, 25, 50, 100 µM) and used distilled water (0 µM SeNPs) as the control for a duration of 12 h to ensure adequate absorption of the solution by the seeds. The pre-sowing seed treatment known as “seed priming” enables seeds to assimilate sufficient water to initiate pre-germinative metabolic activities, but not to induce radical prominence. Furthermore, it aids in the softening of the rigid seed coat. Various enzymes responsible for the mobilization of dietary reserves become bioactive [39]. The soaked seeds were subsequently sown in a controlled growth chamber located in the plant physiology lab of the Faculty of Science at Helwan University. The seeds were placed in small plastic pots filled with loamy soil and were irrigated with tap water up to 70% of the field capacity. Following a period of 15 days, samples were gathered to assess growth parameters such as shoot length, root length, fresh and dry seedling weight. Additionally, chemical analysis was conducted to determine the presence of soluble sugars and proteins.

### Total soluble sugars

0.1 g of freshly picked leaves was extracted using 5 ml of 70% ethanol. The supernatant was then dehydrated using centrifugation until it reached a certain volume. The anthrone technique, as described by [40], was utilized to ascertain the total sugar content. Three milliliters of an anthrone solution, containing two grams per liter of 95% H_2_SO_4_, were combined with one milliliter of a plant extract sample. The resulting mixture was placed in a boiling water bath for 3 min. The color generated was measured at a wavelength of 620 nm via spectrophotometry following the reduction of temperature.

### Total soluble proteins

A quantity of 0.1 g of newly collected leaves was extracted using 5 mL of 70% ethanol. The supernatant was subsequently centrifuged to achieve a known volume. To quantify the soluble protein content, a new solution comprising 5 mL of a 2% sodium carbonate solution in 0.4% sodium hydroxide and 0.5% copper sulfate in 1% sodium tartrate was combined with 1 mL of the extract, yielding a volume (v/v) ratio of 50:1. After a ten-minute reaction period, the mixture was adjusted to a precise volume by adding 0.5 mL of Folin phenol reagent in a 1:1 ratio. Following a 30-minute interval, the optical density of the mixture was measured at 750 nm [41].

### Docking study

Molecular docking of selenium nanoparticles (SeNPs) was conducted using MOE (2009) against five protein targets—*E. coli* RNA polymerase (3LU0), *S. aureus* RNA polymerase (4EDG), *C. albicans* CYP51 (5FSA), superoxide dismutase (2SOD), and auxin receptor TIR1 (2P1Q)—selected for their roles in bacterial transcription, fungal sterol biosynthesis, antioxidant activity, and plant hormone signaling, respectively, with protein structures prepared by adding hydrogens and calculating charges using Amber10:EHT, SeNPs treated as flexible ligands from PubChem, and top poses scored based on binding affinity, hydrogen bonding, and key residue interactions to provide insights into potential mechanisms underlying SeNP biological functions.

### Statistical analysis

Each experimental results is reported as the mean of three replicates with ± a standard deviation. An analysis of statistical significance was conducted comparing samples using IBM SPSS Statistics 21 software. An analysis of variance using one-way ANOVA and Duncan’s multiple range tests were conducted at a significant level of *p* < 0.05.

## Results

### Characterization of biosynthesized nano-selenium

**Fig. 1:**
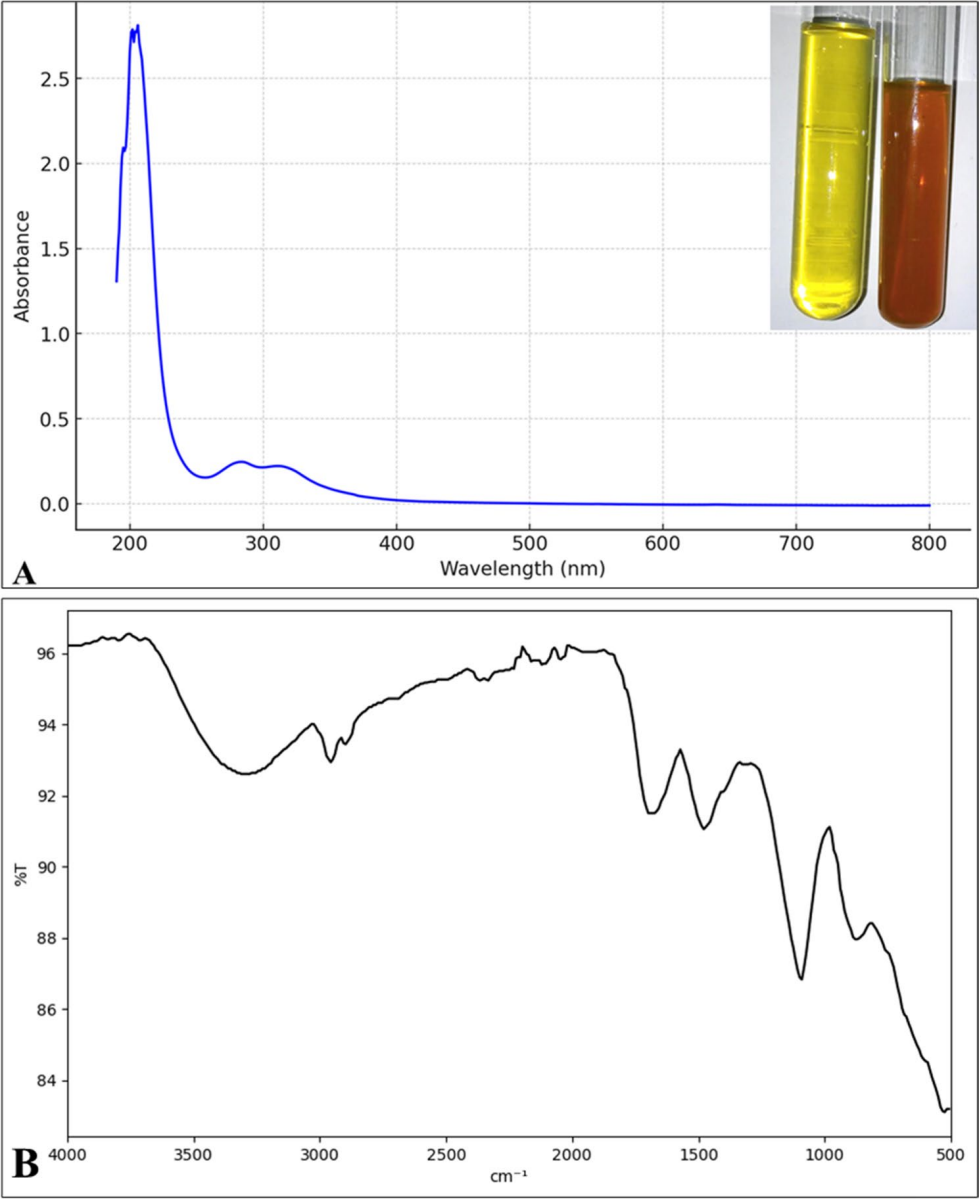
Biosynthesized Nano-selenium (**A**) UV–visible absorbance spectrum analysis of plant SeNps and extract and Color of SeNPs at 24 h (**B**) FT-IR analysis

**Fig. 2:**
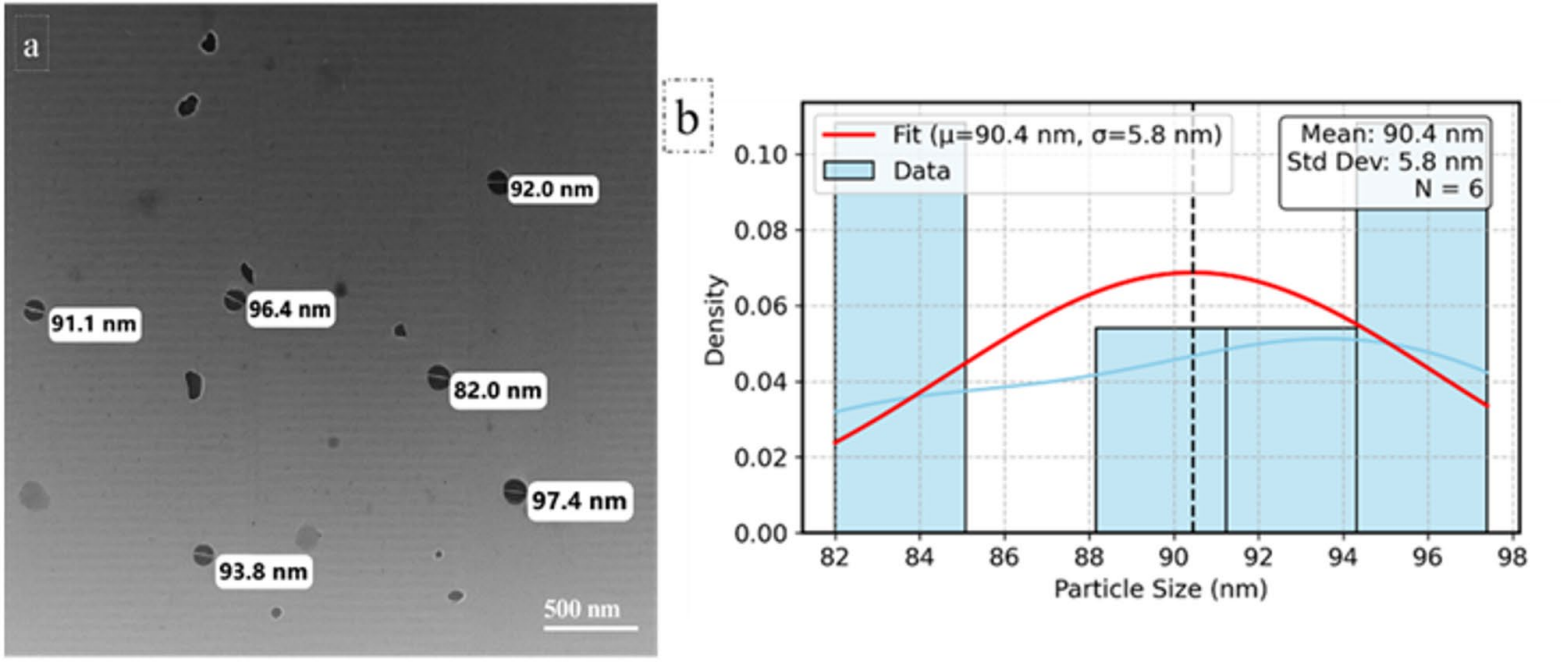
**a** Characterization of the synthesized SeNPs using Transmission Electron Microscope (TEM) and (**b**) particle size distribution of SeNPs from TEM analysis

**Fig. 3:**
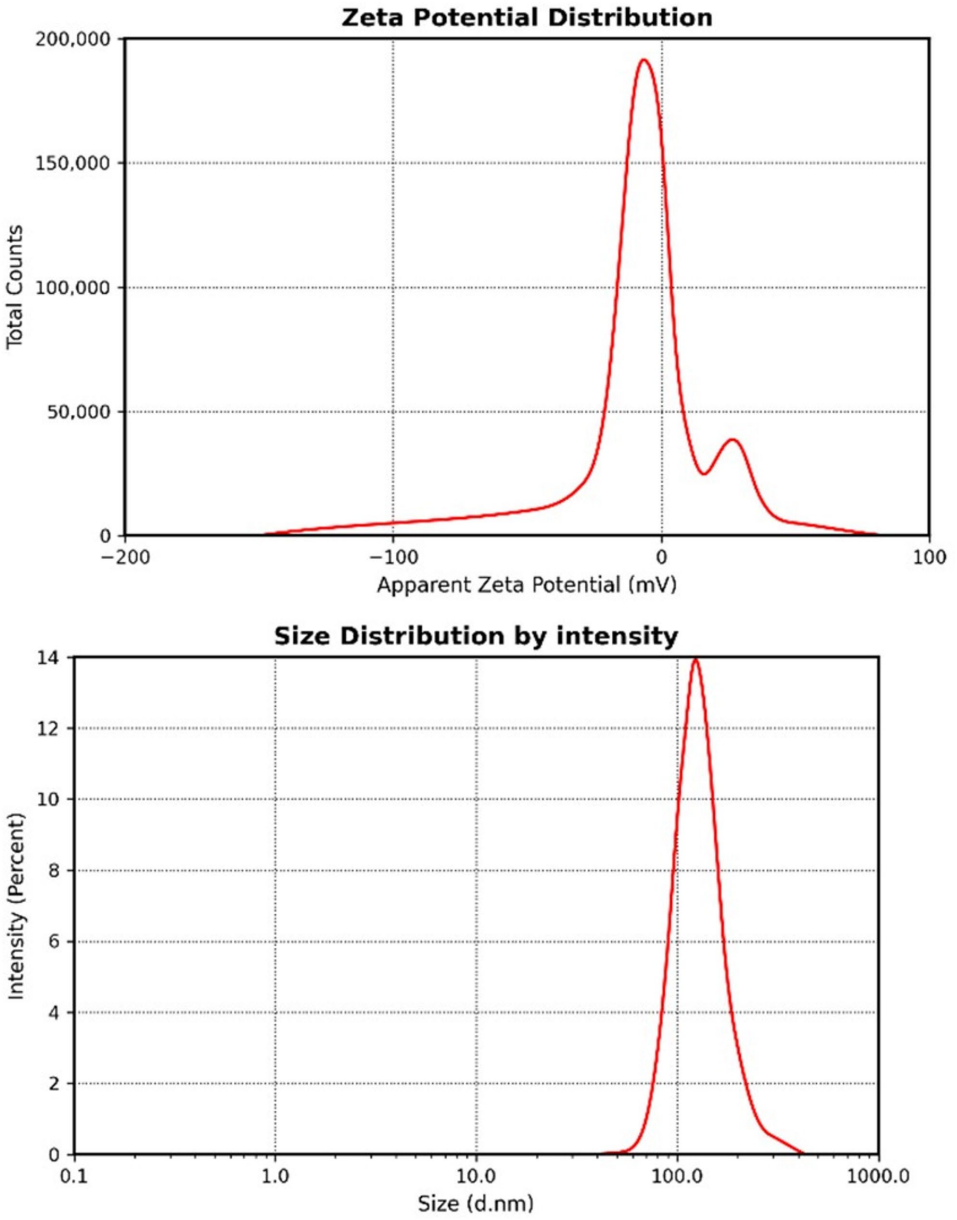
Characterization of selenium nanoparticles, size and surface charge of biosynthesized Nano-selenium by zeta sizer and zeta potential

In (Fig. 1A), besides color change to dark orange, the synthesis of Se-NPs was proved by measuring at a wavelength between 200 and 800 nm. UV-Vis spectroscopy revealed the absorbance peak at 220 nm which indicates the synthesis of selenium nanoparticles. Figure (1B) indicated the presence of several bands with sight shifting wave numbers in the FTIR spectra of selenium nanoparticles. These bands were observed at wave numbers 3206.55, 2919.09, 2960.36, 1566.75, 1403.13, 1027.43, and 418.83 cm^−1^. The bands correspond to phenolic OH, aromatic in-plane C-H bending, asymmetric C-H bending, and secondary OH, respectively, at wave numbers 1375 cm^−1^, 1030 cm^−1^, 1462 cm^−1^, and 1250 cm^−1^. Identified peaks at 2840 and 2930 cm^−1^, Analysis of the SeNPs using FTIR reveals a prominent peak at 3450 cm^−1^. The OH peak may originate from hydroxyl groups present in alcohols, phenols, or carboxylic acids present in the extract. The transmission electron microscopy (TEM) image of SeNPs positively verifies the particles’ spherical form and their even dispersion, with an average diameter of 90.4 nm (Fig. 2). The hydrodynamic diameter obtained by the DLS technique indicated a particle size distribution of around 125 nm and a zeta potential of–ve 10mv (Fig. 3).

### The antioxidant activity

**Fig. 4:**
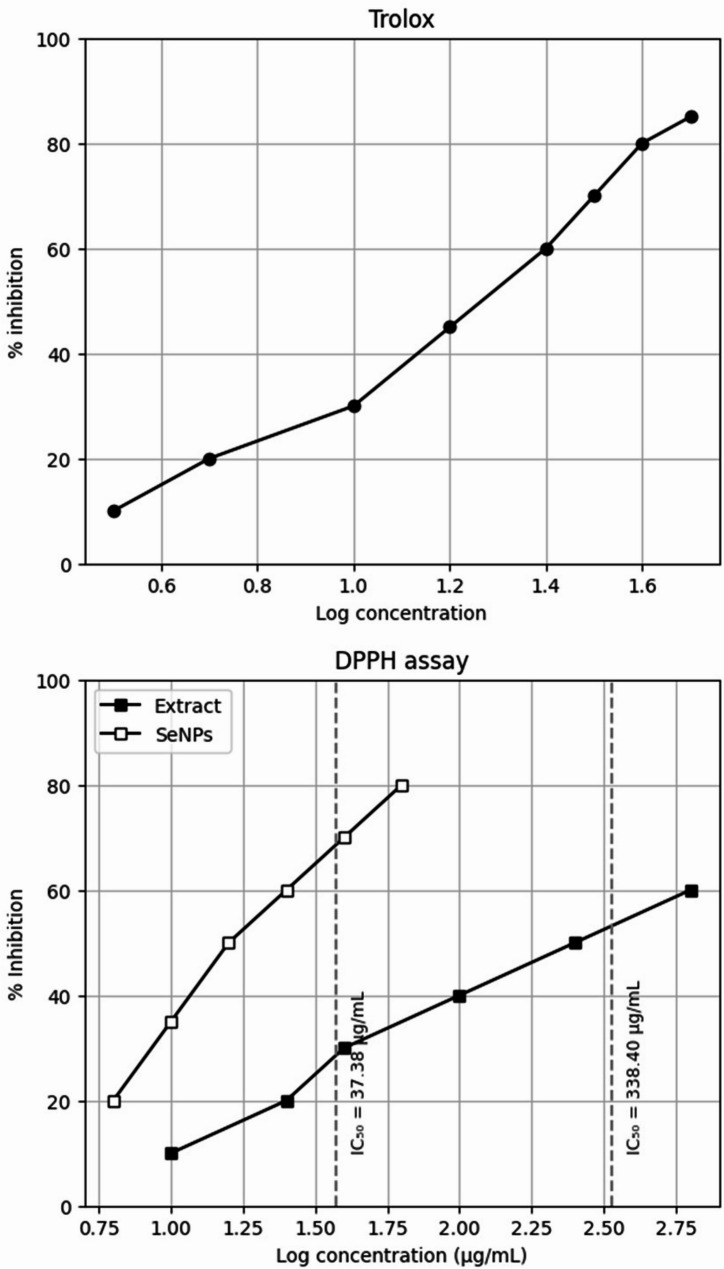
Antioxidant activity of biosynthesized Nano-selenium and the Ferula asafetida Plant extract using DPPH method assay

The antioxidant capacity of plant extract and SeNPs was evaluated by the 2,2-diphenyl-1-picrylhydrazyl (DPPH) test. The findings (Fig. 4; Table 1) revealed that the aqueous extract of *Ferula* exhibited a significant antioxidant activity, with IC_50_ values of 37.38 µgml^−1^. This was followed by the synthesis of SeNPs by *Ferula* extract, which presented an IC_50_ value of 338.4 µgml^−1^.

**Table 1:** Antioxidant activity, IC_50_ determination of biosynthesized senps and *Ferula asafetida* extract

Samples	IC_50_ (µg/mL) (Mean ± SE)
Standard Trolox	6.541 ± 0.21
Biosynthesized Nano-selenium	338.4 ± 1.09
Plant extract	37.38 ± 1.04

### The antimicrobial activity

The antibacterial effectiveness of the plant extract and the SeNPs produced by green synthesis was examined against two bacterial strains (*E. coli* and *S. aureus*) and one fungus strain (*C. albians*). The *Ferula* extract exhibited the greatest zones of inhibition against *C. albicans* (17 mm), but SeNPs showed the strongest activity against *E. coli* (18 mm) and *S. aureus* (15 mm) according to the results presented in (Table 2).

**Table 2:** Antimicrobial activity of biosynthesized senps and *F. assa-foetida* plant extract

Treatments	Inhibition zone diameter (mm)		
	Staphylococcus aureus ATCC25923	Escherichia coli ATCC25922	Candida albicans ATCC1031
Biosynthesized Nano-selenium	15 ± 1.04^a^	18 ± **0**.2^a^	13 ± 0.1^c^
**Plant** **extract**	13 ± 1.08^c^	15 ± 1.01^b^	17 ± 1.07^a^
Vancomycin (20 µg/mL)	16 ± 0.1^b^	Nt	Nt
Gentamicin (20 µg/mL)	Nt	18 ± 0.1^a^	Nt
Amphotericin B (20 µg/mL)	Nt	Nt	14 ± 0.3^b^

The inhibition zone area data, measured as the length of the clear zone around the well, were presented as means ± SD from three repetitions. Means within the same column indicated by distinct letters demonstrate significant differences (*P* < 0.05). The well’s diameter measures 5 mm. Nt: untested

### Impact of Se-NPs on seedling growth criteria of ***V.faba*** (L.)

**Fig. 5:**
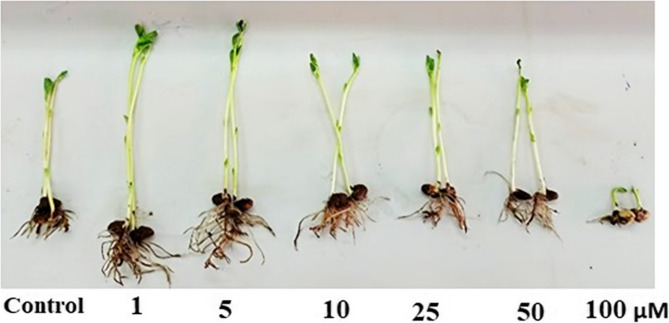
Effect of different concentrations of biosynthesized Nano-selenium (μM) on seedling growth of Vicia faba (L.) (15 days old)

Results represented in (Table 3; Fig. 5) showed that SeNPs at low concentrations 1 µM and 5 µM increased *V. faba* (L.) seedling shoot (23.5, 19.5 cm) and root length (8.5, 6.5 cm) compared to control shoot and root length (15.0, 4.0 cm). SeNPs also improved seedling fresh and dry weight at especially 1 µM by 15.45 and 2.45 g respectively compared to corresponding control (8.89 and 1.32 g). However, at higher concentrations (50, 100 µM), a decrease in seedling growth was recorded.

**Table 3:** Effect of different extracts of biosynthesized Nano-selenium (µM) on morphological characters of *Vicia faba* plant (15 days old) and the soluble sugars, proteins contents (mg g^−1^f.m). Data shown in the table represent the mean ± standard error, followed by a small letter; similar letters indicate that means were not different significantly at 5%, probability based on duncan’s test

Biosynthesized Nano-selenium µM	Shoot length. cm	Root length cm	Seedling fresh weight (g)	Seedling dry weight (g)	Soluble sugars	Soluble proteins
0.0	15.0 ± 0.39 cd	4.0 ± 0.15 d	8.89 ± 0.23 d	1.32 ± 0.084 bc	73.44 ± 1.92 d	281.54 ± 7.32 d
1	23.5 ± 0.61 a	6.0 ± 0.26 b	15.45 ± 0.45 a	2.45 ± 0.12 a	115.35 ± 3 a	374.74 ± 9.75 a
5	19.5 ± 0.55 b	8.5 ± 0.30 a	12.21 ± 0.38 b	2.02 ± 0.11 ab	88.18 ± 2.29 b	340.52 ± 8.86 b
10	16.0 ± 0.50 c	6.5 ± 0.28 b	10.77 ± 0.33 c	1.83 ± 0.093 ab	81.83 ± 2.13 c	314.64 ± 8.19 c
25	15.5 ± 0.45 cd	5.1 ± 0.19 c	9.56 ± 0.29 d	1.77 ± 0.091 ab	74.73 ± 1.94 d	301.01 ± 7.83 cd
50	14.0 ± 0.35 d	4.0 ± 0.13 d	6.32 ± 0.18 e	**0.911** ± 0.07 cd	59.79 ± 1.55 e	248.99 ± 6.47 e
100	4.0 ± 0.10 e	2.5 ± 0.063 e	2.55 ± 0.09 f	0.520 ± 0.053 d	43.76 ± 1.13 f	133.95 ± 3.48 f
LSD at 5%	1.7	0.9	1.25	0.69	6.3	25.9

### Impact of Se-NPs on total soluble sugars and proteins of ***V. faba*** (L.)

**Fig. 6:**
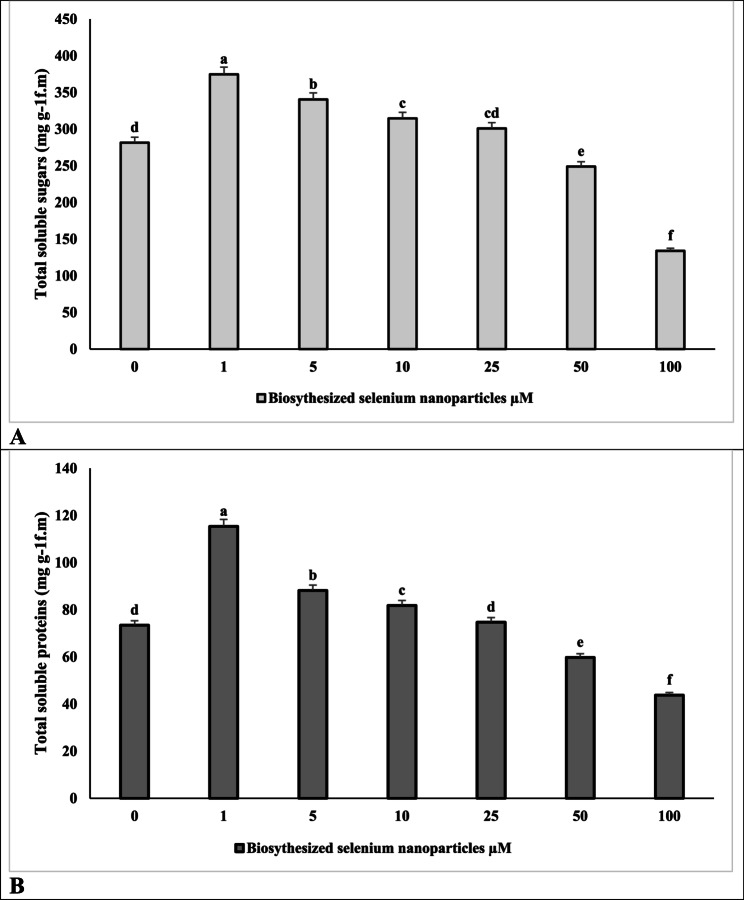
Effect of different concentrations of biosynthesized Nano-selenium (μM) on (**a**) total soluble sugars and (**b**) total soluble protein of Vicia faba (L.). Values represent the mean of three replicates. Different letters (a, b, c, d, e and f) indicate statistical differences at 5% probability according to Duncan’s test. Error bars are standard errors of the mean

Primary metabolites including total soluble sugars and proteins (Table 3; Fig. 6: a, b) increased significantly by application of SeNPs at low doses (57.06 and 33.1% at 1 µM concentration) compared to corresponding control. However, by increase in concentration up to 100 µM total soluble sugars decreased by 40.41% and total soluble proteins decreased by 52.42% compared to corresponding control content.

### Docking study

**Fig. 7:**
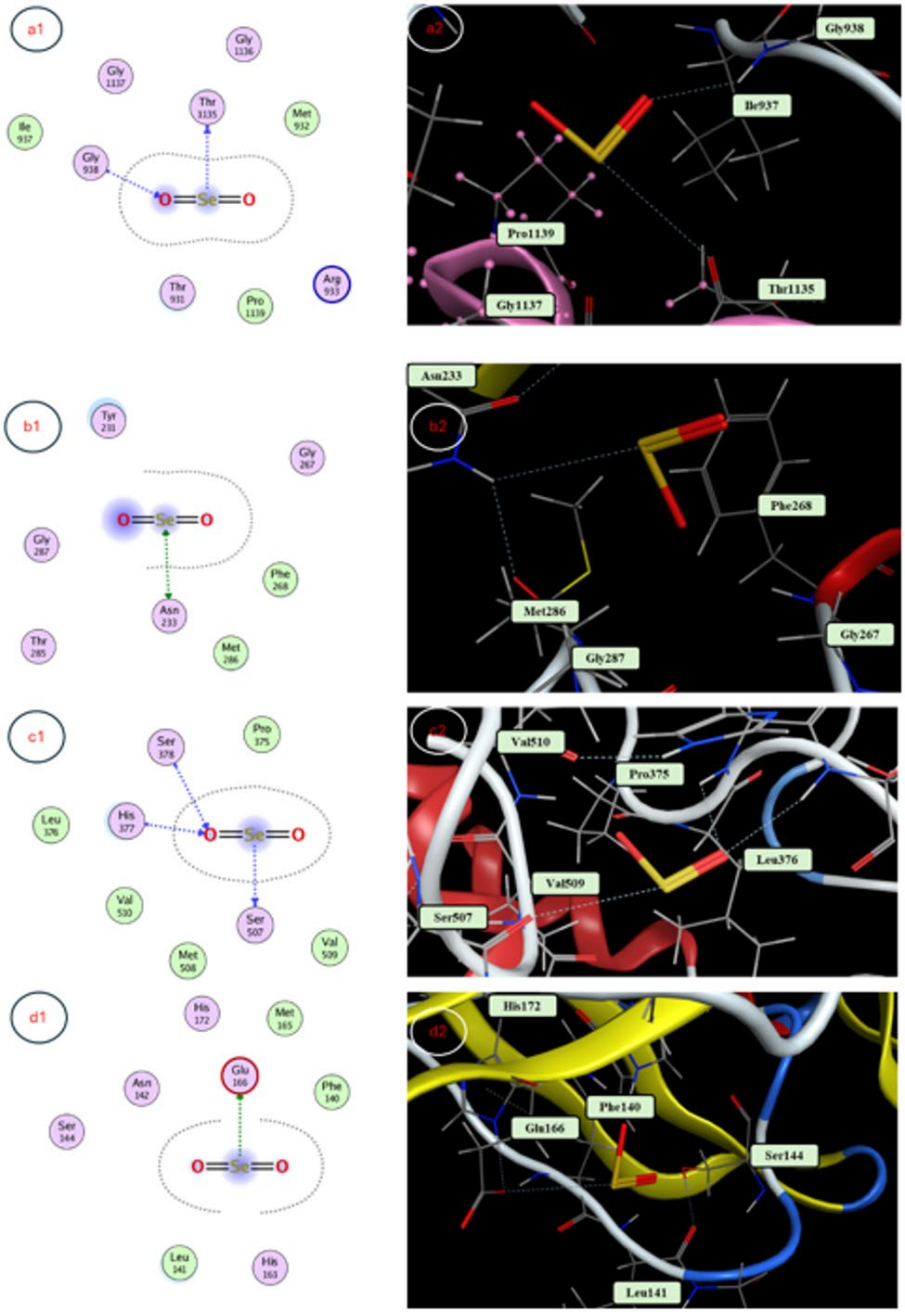
Molecular docking interaction maps depicting 3D and 2D poses of SeNPs docked within active sites of key enzymatic targets: (a1) E. coli RNA polymerase, (b1) S. aureus RNA polymerase, and (c1) C. *albicans* CYP51, yielding insights into potential multifactorial mechanisms of SeNPs activity modulation

**Fig. 8:**
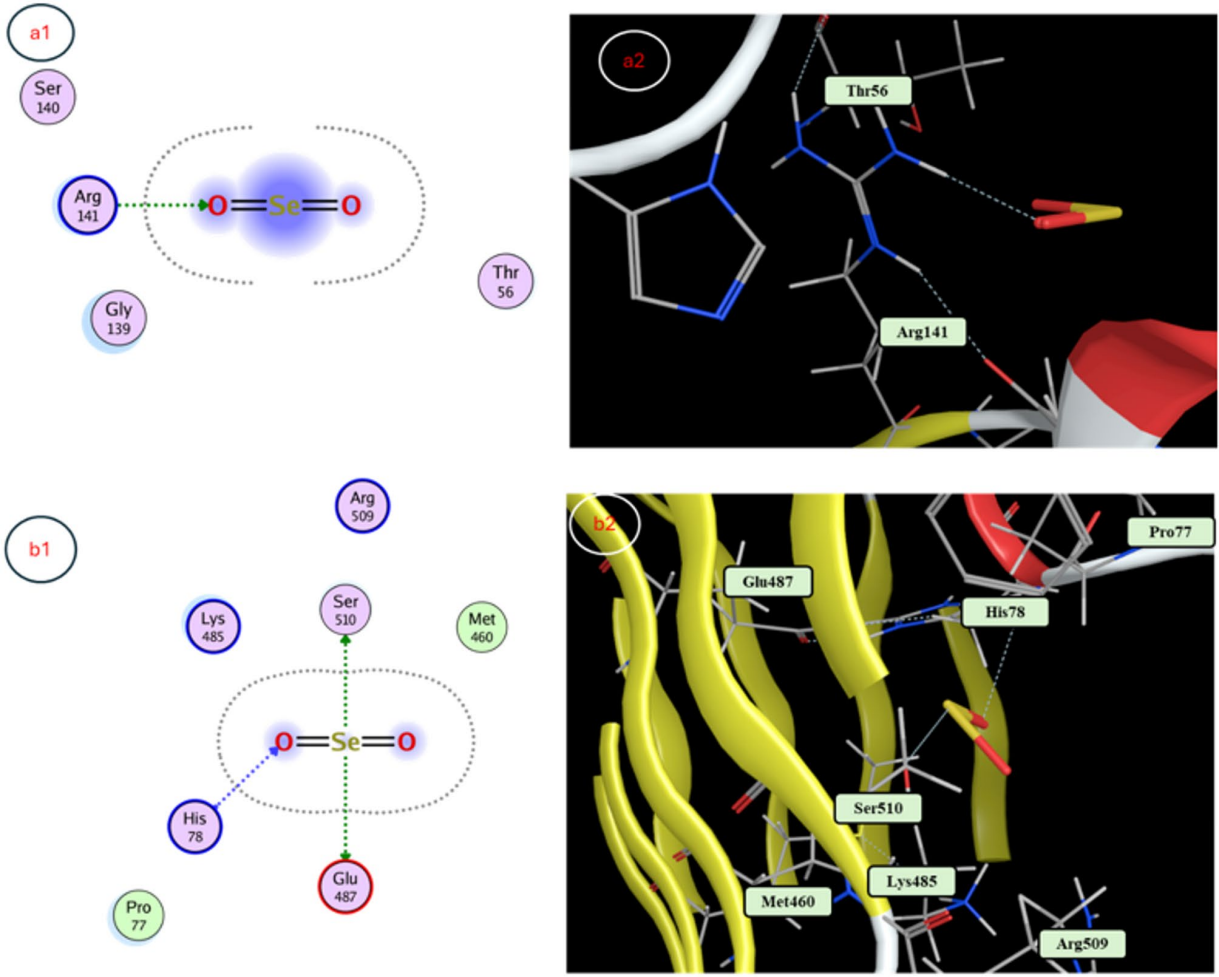
Molecular docking interaction maps providing visualization of SeNPs binding. Depicted are 3D and 2D poses from docking SeNPs within the active sites of: (a1) the antioxidant enzyme superoxide dismutase, and (b1) the plant hormone auxin receptor TIR1, offering insights into how SeNPs may modulate redox homeostasis and phytohormonal signaling in mediating pleiotropic effects

The molecular docking revealed SeNP binding to *E. coli* RNA polymerase (−3.3224 kcal/mol, Thr1135, Gly938, supporting 18 mm inhibition zone), *S. aureus* RNA polymerase (−3.0732 kcal/mol, Asn233, 15 mm inhibition zone), *C. albicans* CYP51 (−3.5097 kcal/mol, His377, Ser378, Ser507, 13 mm inhibition zone), SOD (−2.0912 kcal/mol, Arg141, antioxidant potential), and TIR1 (−3.3108 kcal/mol, Glu487, Ser510, His78, growth enhancement), indicating stable interactions driving bioactivity (Table 4; Figs. 7 and 8).

**Table 4:** Molecular docking reveals binding poses of senps in active sites of targets implicated in diverse biological mechanisms

Protein Target	Docking Score (Kcal/mol)	Interaction Type	Amino Acid Residue
*E. coli* RNA polymerase	−3.3224	Backbone doner	Thr 1135
		Backbone acceptor	Gly 938
*S. aureus* RNA polymerase	−3.0732	Side chain doner Side chain acceptor	Asn 233
*C. albicans* CYP51	−3.5097	Backbone acceptor	His 377
		Backbone acceptor	Ser 378
		Backbone doner	Ser 507
	−2.0912	Side chain acceptor	Arg 141
Auxin receptor TIR1	−3.3108	Side chain doner	Glu 487; Ser 510
		Backbone acceptor	His 78

## Discussion

Nanotechnology has emerged as a transformative field with immense potential across various sectors, particularly in agriculture and medicine, due to its ability to manipulate materials at the nanoscale for enhanced functionality. Utilizing biological sources for nanomaterial synthesis offers significant advantages, including eco-friendliness, cost-effectiveness, and biocompatibility compared to chemical or physical methods [42]. In agriculture, nanoparticles derived from biological sources, such as plant extracts or microorganisms, can act as plant stimulants, promoting growth, enhancing nutrient uptake, and improving stress tolerance in crops [43]. Additionally, these nanomaterials exhibit potent antimicrobial properties, effectively combating plant pathogens and reducing reliance on harmful chemical pesticides [44]. Their high surface area-to-volume ratio and unique physicochemical properties enable targeted delivery and controlled release, maximizing efficacy while minimizing environmental impact [45]. This multifaceted role of bio-derived nanotechnology underscores its potential to revolutionize sustainable agricultural practices and combat microbial resistance, paving the way for innovative solutions in food security and environmental health [46].

The present study explores the bioactivity of selenium nanoparticles (SeNPs) synthesized using *F. assa-foetida* L. root extract, integrating green synthesis, antimicrobial screening, antioxidant evaluation, *V. faba* (L.) biostimulation, and molecular docking to address the growing need for eco-friendly bioactive agents in medicine and agriculture. Unlike commonly studied plants for SeNP synthesis, such as *A. sativum* or *L. barbarum*, *F. assa-foetida*’s root extract, rich in sulfur compounds, phenolics, and flavonoids, offers a novel approach due to its underutilized phytochemical profile, which enhances SeNP stability and efficacy [47]. This integrated experimental-computational framework distinguishes the study from prior plant-mediated SeNP research, providing an exploratory platform to investigate multifaceted bioactivity while addressing the demand for sustainable nanotechnology applications [48].

Green synthesis of SeNPs using *F. assa-foetida* root extract leverages the plant’s high reducing power, attributed to its elevated phenolic, flavonoid, and carbohydrate content. The extract facilitated the reduction of selenite ions to SeNPs, confirmed by a UV absorbance peak at 220 nm, consistent with reported nano-selenium absorption at 222 nm [49]. This peak may be attributed to the impact of the surface plasmon resonance of the SeNPs [50, 51]. The resulting SeNPs exhibited an average size of 90.4 nm The diameters obtained from DLS (125 nm) and TEM (90.4 nm) differed due to DLS measuring the hydrodynamic size, while TEM assesses the solid core [50, 52]. The results was comparable to fungal-mediated SeNP synthesis using *Penicillium chrysogenum*, which produced smaller (3–15 nm) SeNPs with a 262 nm peak, attributed to microbial metabolites [53]. This eco-friendly approach aligns with recent advances in nanotechnology, offering a safer alternative to chemical synthesis while capitalizing on *Ferula*’s unique phytochemicals to produce bioactive nanoparticles [54]. The negative charge of the produced nanoparticles (−10 mV zeta potential) reflects the electrostatic stability, likely due to *Ferula*’s sulfur compounds acting as capping agents, as noted in similar plant-mediated syntheses [55–57].

FTIR bands were observed at wave numbers 3206.55, 2919.09, 2960.36, 1566.75, 1403.13, 1027.43, and 418.83 cm^−1^. The bands correspond to phenolic OH, aromatic in-plane C-H bending, asymmetric C-H bending, and secondary OH, respectively, at wave numbers 1375 cm^−1^, 1030 cm^−1^, 1462 cm^−1^, and 1250 cm^−1^. Identified peaks at 2840 and 2930 cm^−1^, which corresponded to ether-methoxy-OCH3 groups, suggested the presence of biopolymer lignin linked to SeNPs [58]. Analysis of the SeNPs using FTIR reveals a prominent peak at 3450 cm^−1^. The observed peak is associated with the stretching bonds of O–H, which may originate from hydroxyl groups present in alcohols, phenols, or carboxylic acids [59]. The peak observed at 1,633 cm^−1^ may be ascribed to either the stretching of C═C bonds in unsaturated carboxylic acids or the stretching of C═O bonds. Furthermore, the 1,392 cm^−1^ band could suggest the presence of C–N stretching [60].

The antimicrobial activity of *Ferula assa-foetida* root extract and its derived SeNPs was evident against *E. coli* (18 mm inhibition zone), *S. aureus* (15 mm), and *C. albicans* (13 mm), this may be due to the extract’s sulfur compounds and phenolics likely disrupt microbial cell integrity, while SeNPs enhance this effect by generating reactive oxygen species (ROS), damaging lipids, proteins, and DNA, as reported by Devi et al. [61]. This mechanism is supported by studies on *Cassia javanica*-derived SeNPs, which showed MIC values of 62.5–500 µg/ml against similar pathogens, attributed to cell wall penetration and ATP inhibition [62]. SeNPs’ small size enables penetration of cell walls, inhibiting cellular division and ATP production, aligning with findings by Ahmed et al. [63]. For *C. albicans*, SeNPs may disrupt ergosterol biosynthesis, as suggested by Nile et al. [31], contributing to the observed antifungal activity. These results underscore *Ferula*-mediated SeNPs’ potential as antimicrobial agents, with mechanisms warranting further exploration. Webster et al. [64] reported that SeNPs have significant bacteriostatic effects against both Gram-positive and Gram-negative bacteria, such as *S. aureus*, *Staphylococcus epidermidis*, *Pseudomonas aeruginosa*, and *E. coli*. Guisbiers et al. [65] investigated the effects and potential mechanism of SeNPs in suppressing *C. albicans* biofilms. They found that SeNPs, once attached to the biofilm, might penetrate the pathogen and induce structural disruption by substituting the cell wall with Sulphur. This led to a 50% decrease in the development of biofilms by *C. albicans* when exposed to low concentrations of SeNPs.

Fertilizers play a crucial role in enhancing agricultural productivity and crop yields, making a substantial contribution to food security in less developed countries along with the use of SeNPs in the Selenium fertilization sector [66]. Selenium (Se) is a micronutrient widely recognized for its impact on several physiological and biochemical properties of plants, therefore establishing its significance as a nutrient. Selenium has been identified as a natural agent that enhances the antioxidant activity in plants [67]. The antioxidant capacity of *F. assa-foetida* root extract (IC50: 37.38 µg/mL) surpassed that of SeNPs (IC50: 338.4 µg/mL), reflecting its high phenolic and flavonoid content, which supports its role in SeNP stabilization [47]. In *V. faba* (L.), low SeNP concentrations (1–5 µM) significantly enhanced shoot length (56.6%), root development, soluble sugars (57.06%), and proteins (33.1%), consistent with nano-selenium’s role in activating hydrolytic enzymes like α-amylase and protease via gibberellin signaling [68]. These effects mirror findings in cowpea and kidney bean at low doses [37]. Conversely, high concentrations (50–100 µM) reduced growth, likely due to ROS-induced oxidative stress, as reported in ryegrass and kidney bean [37]. Li et al. [69] have shown that the application of SeNPs to celery leaves substantially increases its concentrations of vitamin C, total flavonoids, total phenols, and total antioxidant capacity. By modulating the β-linolenic acid pathway, selenium polyphenols (SeNPs) were shown to augment the nutrient content in celery and improve its antioxidant capacity, therefore enhancing the nutritional quality of crops. Ahmad et al. [70] field experiment revealed the potentiality of Se-NPs to enhance the growth parameters, chlorophylls, carotenoid, carbohydrates, proteins, phenolic compounds, and free proline content in sunflower, especially at 20 ppm. Selenium’s presence in glutathione peroxidase enhances antioxidant defenses at low doses, regulating ROS levels, while high doses disrupt metabolic activities [71]. These concentration-dependent effects highlight *Ferula*-mediated SeNPs’ biostimulant potential, modulated by physiological and biochemical pathways. Selenium plays a crucial role in plants by regulating cellular structure and function via specific physiological processes. They promote the development of shoots and roots, improve the water saturation of bigger plants, facilitate the absorption of nitrogen, and protect plants from pests and diseases [72]. Yuan et al. [73] identified selenium as a vital factor in enhancing plant growth through its ability to enhance glucose metabolism, chloroplast ultrastructure, accelerate chlorophyll production, and inhibit chlorophyll degradation. At 105 days post-planting, cowpea seeds exposed to foliar treatments of 6.25 and 12.5 M Na_2_SeO_4_ or Se-NPs exhibited elevated levels of total carbohydrate and crude protein compared to the control group. As per the findings of Skrypnik et al. [74], selenium has been shown to enhance the production of secondary metabolites in plants including phenolic compounds derived from amino acid pathways, such as phenylalanine, and modulates overall plant metabolism, encompassing amino acid and protein metabolic processes,. A study conducted by Hussein et al. [66] revealed that the growth of ground nut cultivars was influenced by SeNPs through alterations in photosynthetic pigments, lipid peroxidation, antioxidant enzymes (ascorbic acid peroxidase, catalase, and peroxidase), total soluble sugars, phenol content, and total flavonoids in the plants. Selenium nanoparticles (SeNPs) have the potential to be a valuable remedy for the increasing problem of both abiotic and biotic stresses. The absorption and transport of selenium in crops are analogous to those of phosphate and sulphate [75]. At the cellular level, it can replace Sulphur to synthesize crucial macromolecules such as amino acids, specialized structural and functional proteins, and potentially dangerous non-specific proteins, as well as other selenium compounds [76]. Nano-Se demonstrates superior efficacy in increasing the activity of selenoenzymes and displays lower toxicity compared to selenite [77].

From docking studies to mechanistic insights, our investigation revealed that biosynthesized selenium nanoparticles (SeNPs) exhibit multifunctional biological activities through specific molecular interactions. The molecular docking simulations demonstrated strong binding affinities of SeNPs to key biological targets: bacterial RNA polymerases (*E. coli*: −3.32 kcal/mol; *S. aureus*: −3.07 kcal/mol), fungal CYP51 (−3.51 kcal/mol), the antioxidant enzyme SOD (−2.09 kcal/mol), and the auxin receptor TIR1 (−3.31 kcal/mol). These computational predictions provide a structural foundation for understanding the observed antimicrobial, antioxidant, and growth-promoting effects of SeNPs. The antimicrobial efficacy of SeNPs against *E. coli*, *S. aureus*, and *C. albicans* was empirically confirmed through inhibition zone assays, with particularly strong activity against *E. coli* (18 mm). This aligns with our docking results showing SeNP binding to RNA polymerase subunits, suggesting potential disruption of bacterial transcription machinery [78]. Similarly, the antifungal activity against *C. albicans* (13 mm) correlates with the docking results for CYP51, a critical enzyme in fungal ergosterol biosynthesis. These findings are supported by studies demonstrating that nanoparticle interactions with microbial enzymes like cytochrome P450 (CYP450) can disrupt cell membrane integrity and metabolic pathways [79]. Our antioxidant capacity measurements revealed that while the Ferula extract showed superior free-radical scavenging activity (IC50: 37.38 µg/mL), the SeNPs still exhibited significant antioxidant potential (IC_50_: 338.4 µg/mL). The docking interaction with SOD (−2.09 kcal/mol) suggests a mechanism for this activity, paralleling the function of enzymes like monodehydroascorbate reductase (MDHAR) in maintaining cellular redox balance [80]. This enzymatic perspective helps explain how SeNPs may modulate oxidative stress responses in biological systems. The growth stimulation of *V. faba* (L.) at optimal SeNP concentrations (1–5 µM) showed remarkable improvements in shoot length (+ 56.7%) and root development. Our docking results revealed SeNP binding to the auxin receptor TIR1 (−3.31 kcal/mol), suggesting phytohormonal regulation similar to natural auxins like IAA (−3.5 to −4.2 kcal/mol). This aligns with gene expression studies in other plants where TIR1 activation triggers AUX/IAA degradation and subsequent growth-related gene transcription. The concentration-dependent effects mirror findings in Panax ginseng, where key biosynthetic enzymes like HMGR show dose-responsive activity patterns [81]. Future studies should incorporate including detailed enzymatic assays (e.g., RNA polymerase inhibition kinetics), transcriptomic analysis of auxin-responsive genes, and localization studies to track SeNP-enzyme interactions in planta. This integrated approach, combining computational predictions with experimental validation and enzymatic perspectives, provides a comprehensive understanding of SeNPs. The convergence of docking studies, antimicrobial assays, and growth response data establishes a robust framework for developing SeNP applications in both agricultural and biomedical fields.

## Conclusion

This study demonstrates the successful green synthesis of selenium nanoparticles using *F. assa-foetida* L. root extract, producing stable, spherical nanoparticles with an average size of 90.4 nm and a zeta potential of −10 mV, which exhibited potent antimicrobial activity against *E. coli*, *S. aureus*, and *C. albicans*, while the extract showed superior activity against *C. albicans* and exceptional antioxidant capacity (IC_50_: 37.38 µg/mL vs. SeNPs’ 338.4 µg/mL); low SeNP concentrations (1–5 µM) significantly enhanced *V. faba* (L.) seedling growth, increasing shoot length by up to 56.66%, root length from 4.0 to 8.5 cm, and primary metabolites like soluble sugars (57.06%) and proteins (33.1%), though high concentrations (50–100 µM) inhibited growth; molecular docking revealed SeNP binding affinities of −3.5097 to −2.0912 kcal/mol to bacterial RNA polymerases, fungal CYP51, superoxide dismutase, and auxin receptor TIR1, suggesting mechanisms such as transcription inhibition and auxin-mediated growth promotion, thus establishing *Ferula*-mediated SeNPs as promising eco-friendly antimicrobial agents and agricultural biostimulants, with future research needed to optimize application doses and validate molecular mechanisms in plant.

## Data Availability

Data is provided within the manuscript or supplementary information files.
